# 5G intelligent airport platform for UAV blood transport in Shenzhen China: a retrospective observational study

**DOI:** 10.3389/fpubh.2026.1947820

**Published:** 2026-09-30

**Authors:** Li Ning, Jinhong Liu, Sheng Zhang, Tailong Tan, Liqin Huang, Geng Zhang, Xuqun Wu, Litao Wu

**Affiliations:** 1 Shenzhen Blood Center, Shenzhen, China; 2 Shenzhen Bao'an District Central Blood Station, Shenzhen, China

**Keywords:** 5G-enabled intelligent platform, blood transport, emergency blood supply, Shenzhen, unmanned aerial vehicles (UAVs)

## Abstract

**Background and aims:**

Efficient and secure blood transport is critical in healthcare. Unmanned aerial vehicles (UAVs, drones) offer potential advantages in timeliness and safety, with more than 10 regions in China having implemented drone-based blood transport. However, challenges remain, including limited visual monitoring and management models constrained by specific drone types and operational conditions. Developing an open, collaborative, and intelligently managed drone transport platform is thus of significant value.

**Methods:**

Shenzhen Blood Center utilized 5G technology to enable intelligent closed-loop management of blood distribution, route planning, and emergency dispatch and hospital reception. The system supports real-time temperature monitoring and live video surveillance during flights. By integrating open drone airports and intelligent management systems, the first “5G-enabled Intelligent Airport Platform for Drone Blood Transportation” in China was established, accommodating multiple multi-rotor drone models. Its feasibility and operational effectiveness were systematically evaluated.

**Results:**

As of August 31, 2025, the platform includes two drone systems, 18 routes, and serves 15 hospitals. From January 1, 2024, to August 31, 2025, 11,784 flights transported 8,320.82 kg of blood, including 25,803 U of red blood cells, 3,383 therapeutic doses of platelets, 1,357,733 ml of plasma, and 10,314 U of cryoprecipitate. The cumulative flight time was 178,640.9 min, with an average flight duration of 15.2 min. Compared to ground transport, drones saved an average of 46.8 min (peak) and 32.3 min (off-peak). All blood products met temperature requirements, with no damage or temperature deviations recorded.

**Conclusions:**

This study demonstrates that a 5G-based drone platform reliably supports routine and emergency blood transport, ensuring timeliness and quality. It offers an innovative management model for blood collection and supply institutions. Future efforts will expand routes and coverage, enhance emergency supply capacity, establish a citywide drone blood transportation network and promote the low-altitude economy in healthcare.

## 1 Introduction

As a critical medical resource, the timely and secure delivery of blood remains a significant challenge for blood stations. Clinically, blood transportation is directly linked to whether patients receive timely and effective treatment, emphasizing the need for high timeliness and reliability in blood transport. Traditional ground transportation is often constrained by road conditions, particularly during rush hours, making it difficult to meet the clinical demands for timeliness and reliability, and even potentially leading to medical rescue incidents (1, 2). Therefore, the mode of blood distribution is essential for ensuring the timeliness of medical treatment (3). In emergency scenarios where faster response is required, there is an urgent need for transportation methods capable of rapid response and efficient arrival, a capability currently lacking in ground transportation systems. Thus, given the limited supply of blood, stringent requirements for storage and transportation conditions, short shelf life, and random fluctuations in demand across hospitals, exploring new transportation models is of vital importance in the blood transportation field.

Drones, as core enablers of the low-altitude economy, significantly enhance logistics efficiency and offer innovative solutions for key areas such as emergency response and medical delivery, and some research has indicated the significant value of drone-based blood delivery in reducing time and minimizing waste of blood components (4–6). International experts have discussed three promising innovative strategies for blood transfusions in blood deserts, one of which is drone-based blood delivery (7). Since 2021, blood stations in China have begun exploring the use of multi-rotor small drones for blood transport, showing good timeliness, feasibility, operability, and application prospects for emergency blood distribution (8–10). Drone blood transportation has been implemented in multiple provinces and municipalities, including Guangdong, Yunnan, Zhejiang, Jiangsu, Hubei, Hebei, TianJin, Shandong, Liaoning, Sichuan, Guizhou, Qinghai and so on in China. Given that drones offer advantages such as high speed, enhanced safety, and independence from ground traffic constraints, they have become one of the preferred options for building a sound urban healthcare systems and enhancing public health emergency response capabilities. Additionally, studies have confirmed that drone transportation does not adversely affect blood components or related parameters, ensuring blood safety (11–13). In emergency medical scenarios, a study systematically reviewed existing evidence on drone-based transport of blood products in military and austere environments, demonstrating that with appropriate packaging, temperature control, and operational protocols, both red blood cell concentrates and other blood products maintain clinically acceptable quality (14). Moreover, a successful case of massive transfusion performed on the battlefield using autonomously delivered blood products was reported, demonstrating that this technology can be directly integrated into life-saving clinical practice (15). Recent research reported that drones demonstrate significant potential to enhance the speed, coverage, and coordination of pre-hospital emergency care (16). As such, drone-based blood transport is an effective solution for improving the medical rescue support system and enhancing treatment capabilities.

However, the current management of drone blood transportation faces shortcomings, including inadequate monitoring of the transport process, unable to achieve real-time tracking of entire flights, such as drone location and blood temperature, and a lack of competitive services in certain regions, which hinder its large-scale application. To address these issues, a “5G-enabled intelligent Drone Platform for Blood Transportation” has been developed in Shenzhen, China. This platform utilizes 5G technology to perform visual monitoring of the entire transportation process, including drone positioning, blood environmental parameters, blood temperature monitoring, and live video streaming, to ensure blood quality and suitability. It also introduces an innovative open management model that pioneers multi-drone company services, standardizes takeoff and landing for multiple drone types, fostering healthy competition and promoting drone service development. The platform integrates 5G high-speed network transmission, drone intelligent management systems, and open drone platforms, supports end-to-end standardized management of multiple drone types, routes, takeoff and landing points, and blood product types, facilitating the integration of management and clinical needs, reducing operational barriers, and enhancing emergency support capabilities.

## 2 Objectives and methods

### 2.1 Design and planning of drone flight routes

#### 2.1.1 Drone route selection criteria

The 5G-based intelligent airport platform of multi-rotor drone consists of hardware facilities and software facilities, including drone takeoff and landing sites (Supplementary Figures 1), intelligent dispatch rooms, 5G-based intelligent information management system (Supplementary Figure 2). Besides, each receiving hospital is equipped with a standardized fixed landing platform (Supplementary Figure 4). Routes should avoid densely populated areas, sensitive locations (such as schools and hospitals), high-voltage lines, and other aerial obstacles. Priority is given to straight-line shortest routes to optimize flight time. The routes must strictly adhere to airspace control regulations and allow for prompt response to temporary no-fly notices. Continuous coverage of stable 5G/4G signals throughout the flight is essential to ensure communication and navigation reliability. Flight altitude should be controlled in accordance with the regulations of the Civil Aviation Administration of China and local airspace management guidelines.

#### 2.1.2 Sample selection and randomization

As of August 31, 2025, Shenzhen has opened 18 drone blood transportation routes, covering districts including Futian, Nanshan, Bao'an, Guangming, and Luohu. In this study, we analyzed all routes and transportation frequencies during this period. For the temperature analysis, temperature records were randomly selected, as the temperature during blood transport remains highly stable, and these randomly collected records can adequately represent the overall situation. The temperature records were obtained through stratified random sampling based on multiple dimensions, including time and route, ensuring their representativeness.

### 2.2 Timeliness and quality monitoring of drone blood transportation

#### 2.2.1 Timeliness monitoring of blood transportation

The blood transport follows standard drone blood transportation process (Supplementary Figure 3).

Drone transportation time: depending on the drone type and actual transportation needs, the entire flight time from the drone taking off with the blood from the blood center to landing at the hospital is recorded.

Ground transportation time: the entire time taken for the hospital to dispatch a vehicle to the blood center to pick up the blood and deliver it to the hospital is recorded.

Finally, a comparative analysis is conducted between ground transportation times during peak and off-peak periods and drone transportation times.

#### 2.2.2 Quality monitoring during blood transportation

During drone flights, a 5G gateway, wireless temperature control device, and wireless monitoring cameras are embedded into the blood transport logistics box. The box is a medical-grade, temperature-controlled container. Blood products are packed separately by type and must not be mixed. This allows for the simultaneous transport of blood components requiring different temperature conditions, such as red blood cells (2–10 °C), platelets (20–24 °C), and plasma (below 0 °C). After loading, the flight transport begins. Throughout the drone blood transportation process, the 5G temperature-sensing transmission device monitors the environmental temperature and status of the blood in real time, ensuring its quality and suitability. The temperature data is transmitted back to the platform via the 5G low-altitude network, guaranteeing real-time data transmission and stable feedback. This enables remote monitoring of blood status and allows for necessary dispatch measures. The temperature sensors are attached directly to the surface of the blood product packaging, monitoring the actual temperature of the product rather than the ambient temperature inside the container.

After the flight mission is completed, the full journey temperature record can be downloaded, with a focus on monitoring temperature changes before and after transportation for blood products such as packed red blood cells, concentrated platelets, and fresh frozen plasma.

### 2.3 Analysis and statistics

#### 2.3.1 Study design and data source

This study employed a retrospective observational design. Data were collected from flight mission reports and transportation records documented by Shenzhen blood center between January 1, 2024, and August 31, 2025. All drone transports with flight data were successful flights considering factors such as weather, air traffic control, and route conflict coordination.

#### 2.3.2 Variables and measures

Primary outcome: transport time for drone and ground vehicles in peak and off-peak hours. (measured in minutes).

Secondary outcomes: total number of flights and the volume of various blood components transported (e.g., red blood cells, plasma, platelets and cryoprecipitate).

Grouping Variable: data were divided into three groups based on transport mode: the drone transport group, peak-hour ground transport group, and off-peak ground group.

#### 2.3.3 Statistical analysis

Statistical analysis was performed using Graphpad Prism.

Descriptive Analysis: descriptive statistics were calculated for the frequency of flights and the volume of blood components. Adherence to temperature requirements for suspended red blood cells, platelets, and plasma during the transport process was monitored as an indicator of blood quality.

Inferential analysis: to compare transport efficiency between drones and ground transport. The normality of the data was evaluated using the Shapiro-Wilk test, and the homogeneity of variances was assessed using Bartlett's test. Since the data violated these assumptions (*P* < 0.05), the Kruskal–Wallis test was employed to compare the three independent groups. When a significant overall difference was detected, *post-hoc* pairwise comparisons were performed using Dunn's test with Bonferroni adjustment. All data are presented as median (interquartile range, IQR). A *p*-value of less than 0.05 was considered statistically significant.

### 2.4 Cost and economic evaluation

All the 18 drone routes are rented from company and the cost is the package price which includes all the equipment and personnel fees.

The cost of ground transportation includes the vehicle cost and personnel salary, and the former includes vehicle depreciation, vehicle repair and maintenance cost, fuel or charging cost, insurance, annual inspection cost, and miscellaneous driving fees.

### 2.5 Definition of adverse events

In this study, adverse events specifically refer to the following three situations:

1. The blood temperature exceeds the limit upon delivery, resulting in blood damage;
2. The delivery is too late, affecting clinical use;
3. The blood bag is damaged during transportation.

Minor operational deviations (such as brief delays, adjusting transport methods due to weather, holding in airspace, etc.) are not counted as adverse events, provided that the above three core indicators are not affected.

## 3 Results

### 3.1 Analysis of blood transportation efficiency

From January 1, 2024, to August 31, 2025, a cumulative total of 11,784 flights were conducted, transporting 25,803 U of red blood cells, 3,383 therapeutic doses of platelets, 1,357,733 ml of plasma, and 10,314 U of cryoprecipitate. Monthly analysis of the transportation volumes of various blood components (Table 1) revealed that the maximum transportation volumes of red blood cells occurred in May 2024 while platelets and plasma occurred in October 2024 (Figures 1A–C), while the transportation volume of cryoprecipitate was significantly higher in September 2024 compared to other months (Figure 1D). The cumulative flight time was 178,640.9 min, with an average flight duration of 15.2 min. A comparison of the transportation times of the 18 drone routes with ground transportation times during peak and off-peak hours. Table 2 showed that, during peak hours, drone transportation saved an average of 46.8 min compared to ground transportation, with a maximum saving of 89.3 min. During off-peak hours, drone transportation saved an average of 32.3 min, with a maximum saving of 59.1 min. And both showed significant differences (Figure 2). And we also compared the aerial distance, road distance, approximate road travel time, and UAV travel time between the center and each of the 15 hospitals to better appreciate the comparative time advantage of UAV-based transport. Our result indicated that several UAV deliveries can reduce the total transit time by approximately 60% (Supplementary Table 1).

**Table 1:** Detailed monthly transportation of blood components by drones from January 2024 to August 2025 over a period of 20 months.

No.	Time	Red blood cells (U)	Platelets (U)	Plasma (ml)	Cryoprecipitate (U)
1	Jan 2024	345	85	1,400	0
2	Feb 2024	906	132.5	27,600	50
3	Mar 2024	1,644.5	187.5	57,150	201.5
4	Apr 2024	1283	132	60,500	472
5	May 2024	2,265.5	199	88,050	674
6	Jun 2024	1296	231	84,400	320
7	Jul 2024	1,310.5	99	38,400	360
8	Aug 2024	1,673.5	160.5	66,000	411
9	Sep 2024	1,530.5	217	90,730	3,186
10	Oct 2024	1,988	288	144,500	749
11	Nov 2024	1,810	207	67,000	150
12	Dec 2024	1,555	257	115,550	340
13	Jan 2025	1,647.5	216.5	121,750	1,130
14	Feb 2025	1,487.5	195.5	102,200	690
15	Mar 2025	1,058.5	138	64,500	292
16	Apr 2025	595.5	151.5	37,200	142
17	May 2025	595	102	38,100	114
18	Jun 2025	713.5	80	35,603	500
19	Jul 2025	1,025.5	148	53,400	337.5
20	Aug 2025	1,072.5	156	63,700	195

**Figure 1:**
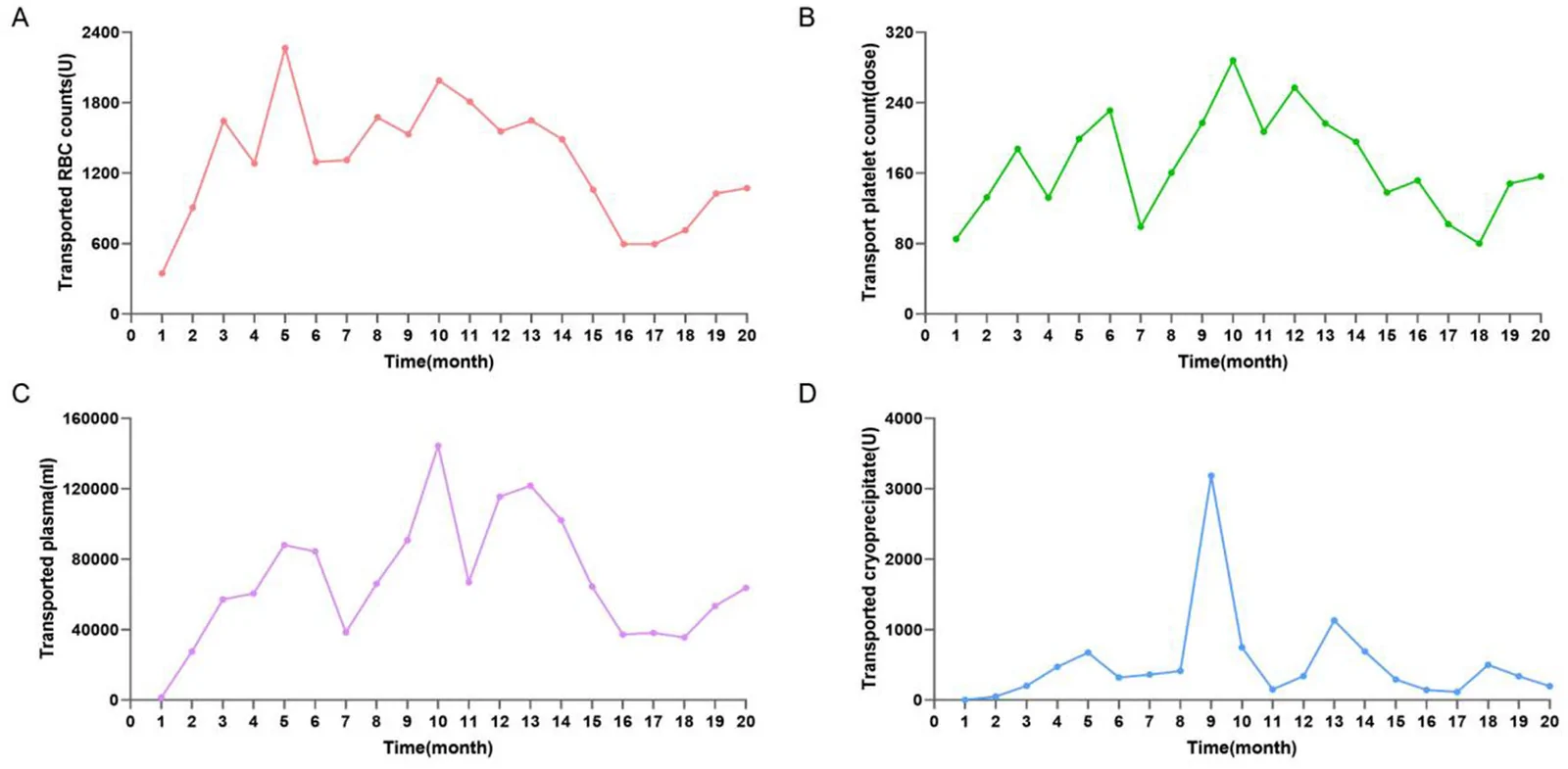
Statistical charts of monthly transportation volumes of various blood products. Statistics of monthly transportation volumes of red blood cells **(A)**, platelets **(B)**, plasma **(C)**, and cryoprecipitate **(D)** by drones from 2024 to August 2025 (20-month period, 1 represents January 2024, 2 represents February 2024, and so on).

**Table 2:** Statistical table of average time for ground and drone transportation of all 18 drone routes.

Route No.	Ground transport time	Ground transport time	Drone	Time saved	Time saved
	(During peak hours)	(During off-peak hours)	Transport time	(During peak hours)	(During off-peak hours)
Route 1	65.7	52.3	19.7	46.0	32.5
Route 2	56.5	37.5	16.7	39.7	20.8
Route 3	54.7	48.2	22.2	32.5	26
Route 4	53.9	41.9	16	37.9	25.9
Route 5	64.1	47.4	18.6	45.5	28.8
Route 6	45.7	38.2	14	31.7	24.2
Route 7	45.1	36.9	13.2	31.9	23.7
Route 8	85.7	78.9	36.1	49.6	42.8
Route 9	128.9	98.7	39.6	89.3	59.1
Route 10	91.8	77	37.7	54.1	39.3
Route 11	84.6	62.9	28.5	56.1	34.4
Route 12	25.8	22.5	5.1	20.7	17.4
Route 13	35.6	30.8	6	29.6	24.8
Route 14	55	39.9	14.9	40.1	25
Route 15	114.2	82.8	35.4	78.8	47.4
Route 16	50.1	41.8	17.2	32.9	24.6
Route 17	127.8	101.3	48.5	79.3	52.8
Route 18	64.6	39.2	10.5	54.1	28.7

The time unit is minutes (min). For the drones in the table whose transportation time exceeds 25 min, including routes 8, 9, 10, 11, 15, and 17, the transportation time includes the stopover time.

**Figure 2:**
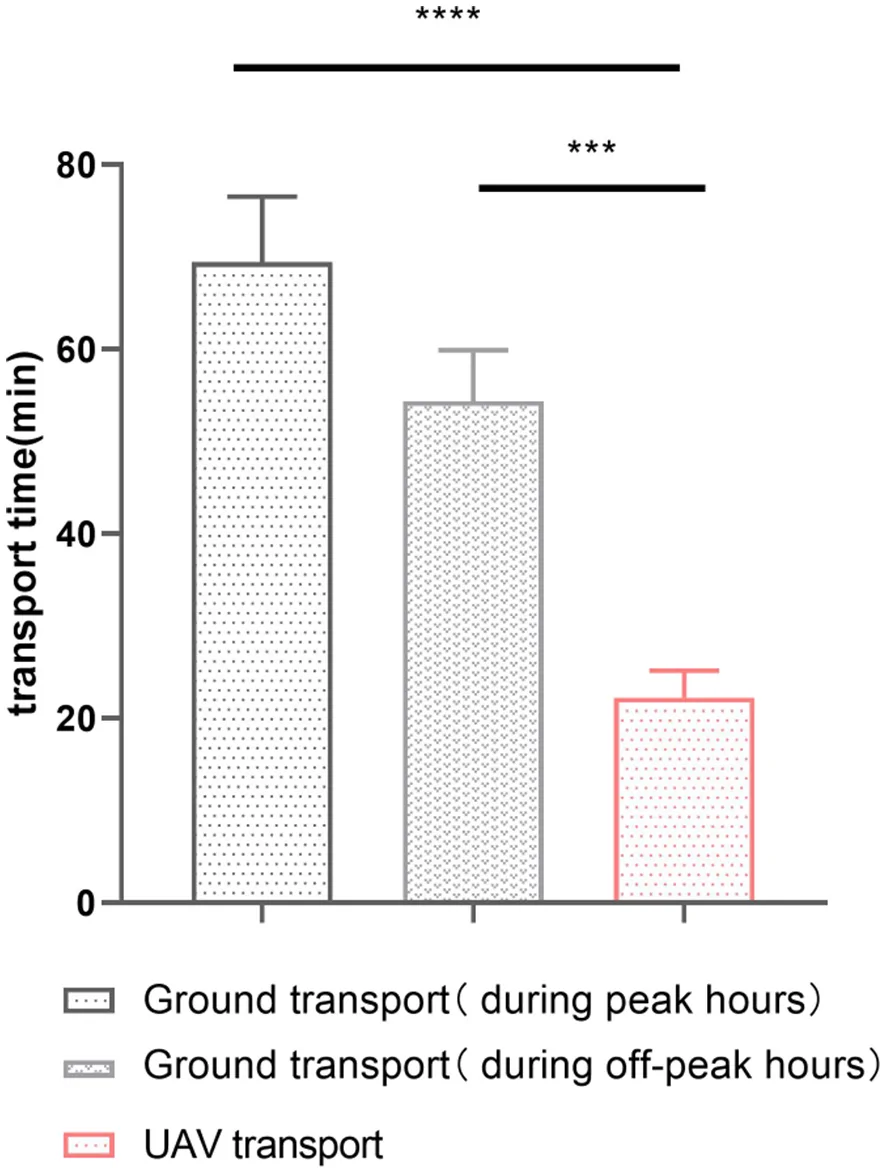
Comparison chart of ground transportation and drone transportation times for 18 routes. The Shapiro–Wilk test indicated that the data in the three groups deviated significantly from a normal distribution (Table 3). Then Kruskal–Wallis test was performed (*H* = 29.5, df = 2, *P* < 0.0001, Table 4), followed by Dunn's multiple comparison test with Bonferroni correction (17). The sample size for each group is 18 (*n* = 18). ****p < 0.0001, ***p = 0.0004.

### 3.2 Analysis of quality and safety of drone blood transportation

For each drone delivery of blood components, we recorded temperature data throughout the entire transportation process to ensure that the products remained within the specified temperature range at all times. We randomly sampled 102 temperature records for RBCs, 105 for platelets, and 29 for plasma for analysis in 2025 (Excel 1). Monitoring data from 30-min flights showed that the temperature of suspended red blood cells remained within 2–10 °C, platelet temperature stabilized between 20–24 °C, and plasma temperature was maintained below 0 °C (Figure 3). All these values comply with the requirements of the Blood Transportation Standard WS 400−2023.

**Figure 3:**
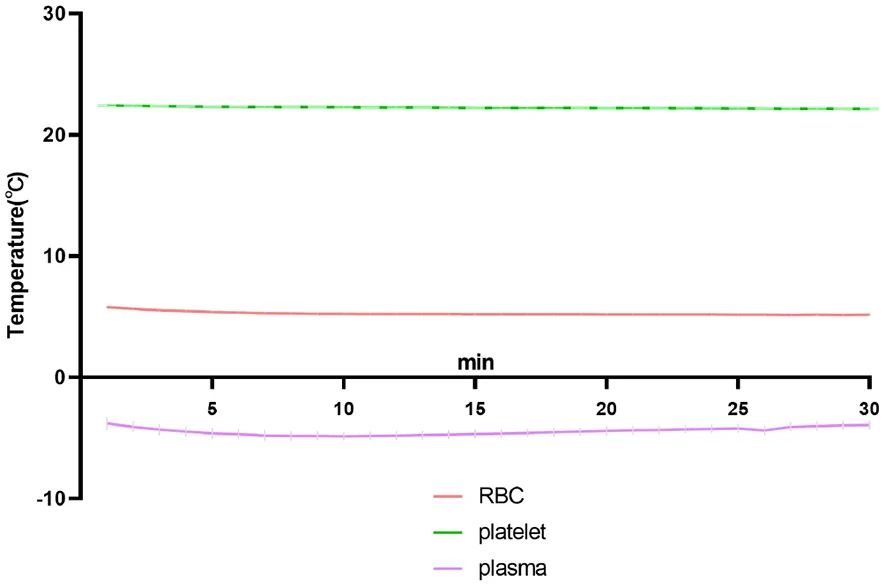
Temperature variation curve during drone transportation of red blood cells, platelets and plasma. The average temperature during transportation of red blood cells was 5.3 °C, with a maximum temperature of 9.1 °C and a minimum temperature of 2.4 °C. The average temperature during transportation of platelets was 22.2 °C, with a maximum temperature of 23.5 °C and a minimum temperature of 20 °C. The average temperature during transportation of plasma was −4.5 °C, with a maximum temperature of 0 °C and a minimum temperature of −9.3 °C.

### 3.3 Comparison of the cost between drone and ground blood transportation

Eighteen drone routes are rented. The total rental for 1 year is 2,700,000 CNY.

For the ground transportation, four blood delivery vehicles have been put into service. The total vehicle cost is about 230,785.61 CNY, the personnel costs is about 600,000 CNY (Table 5). Miscellaneous driving fee is calculated on the transport distance. Assuming the miscellaneous driving fee is X, the total cost of ground transportation per year is”830,785.61+X”CNY. For the ground transport during year 2025, the cumulative transport distance was 162,587 km. At a unit cost of 2.7 CNY/km, the total expenditure was nearly 438,984.9 CNY.

**Table 3:** The data of Shapiro–Wilk test of three groups of ground transport during peak and off peak hours and UAV transport.

Group	*N*	Delivery time (minutes), median (IQR)	Mean ranks	Shapiro–Wilk test (*p*-value)
Ground transport (during peak hours)	18	60.3 (49.05–87.15)	39.11	0.073
Ground transport (during off peak hours)	18	44.7 (38.05–77.45)	31.78	0.0261
UAV transport	18	17.9 (13.8–35.58)	11.61	0.1471

**Table 4:** Pairwise comparisons from Dunn's *post-hoc* test following Kruskal–Wallis test.

Comparision group	Mean rank difference	*Z*-score	Effect size “*r*”	Magnitude of effect	Adjusted *P*-value
A vs. B	39.11–31.78 = 7.33	1.4	0.19	Small (< 0.3)	0.486
B vs. C	39.11–11.61 = 27.50	5.24	0.71	Large (>0.5)	< 0.0001
A vs. C	31.78–11.61 = 20.17	3.85	0.52	Large (>0.5)	0.0004

A: Ground transport (during peak hours)

B: Ground transport (during non-peak hours)

C: UAV transport

The Z-score for each pairwise comparison was calculated as the difference in mean ranks divided by the pooled standard error specific to Dunn's test (SE = 5.244). This SE was derived from the total sample size and group sizes as: √[(54 × 55/12) × (1/18 + 1/18)] = 5.244.

**Table 5:** Ground transportation cost.

Vehicle no.	Vehicle cost (CNY)						Personnel costs
	Annual depreciation	Repair and maintenance	Fuel (charging)	Insurance	Annual inspection	Vehicle mileage fee	
1	9,367.44	8,690.90	22,946.29	2,398.52	600.00	—	150,000
2	18,944.4	16,838.70	30,510.36	2,814.46	600.00	—	150,000
3	18,944.4	21,008.50	30,485.18	2,814.46	600.00	—	150,000
4	9,367.44	7,693.80	22,727.90	2,832.86	600.00	—	150,000
Total	56,623.68	54,231.9	106,669.73	10,860.3	2,400	—	600,000

“—” indicates the fee is calculated on the transport distance.

### 3.4 Future planning for drone blood transport routes in Shenzhen

Currently, Shenzhen has three main blood collection and supply institutions: the Shenzhen Blood Center, Shenzhen Bao'an District Central Blood Station, and Shenzhen Longgang District Central Blood Station. Among the existing 18 drone routes, connectivity has already been established between the Shenzhen Blood Center and Shenzhen Bao'an District Central Blood Station, as well as between Shenzhen Bao'an District and Shenzhen Longgang District Central Blood Station. In the future, plans are in place to further expand the drone route network with the Shenzhen Blood Center at the core, in collaboration with the other two District Central Blood Station. By adding more routes and aerial emergency channels, the network aims to cover *N* medical institutions (where *N* indicates the number) and establish a “1+2+*N*” blood transport system. This system is designed to create a city-wide, highly responsive emergency blood transport aerial network (Figure 4).

**Figure 4:**
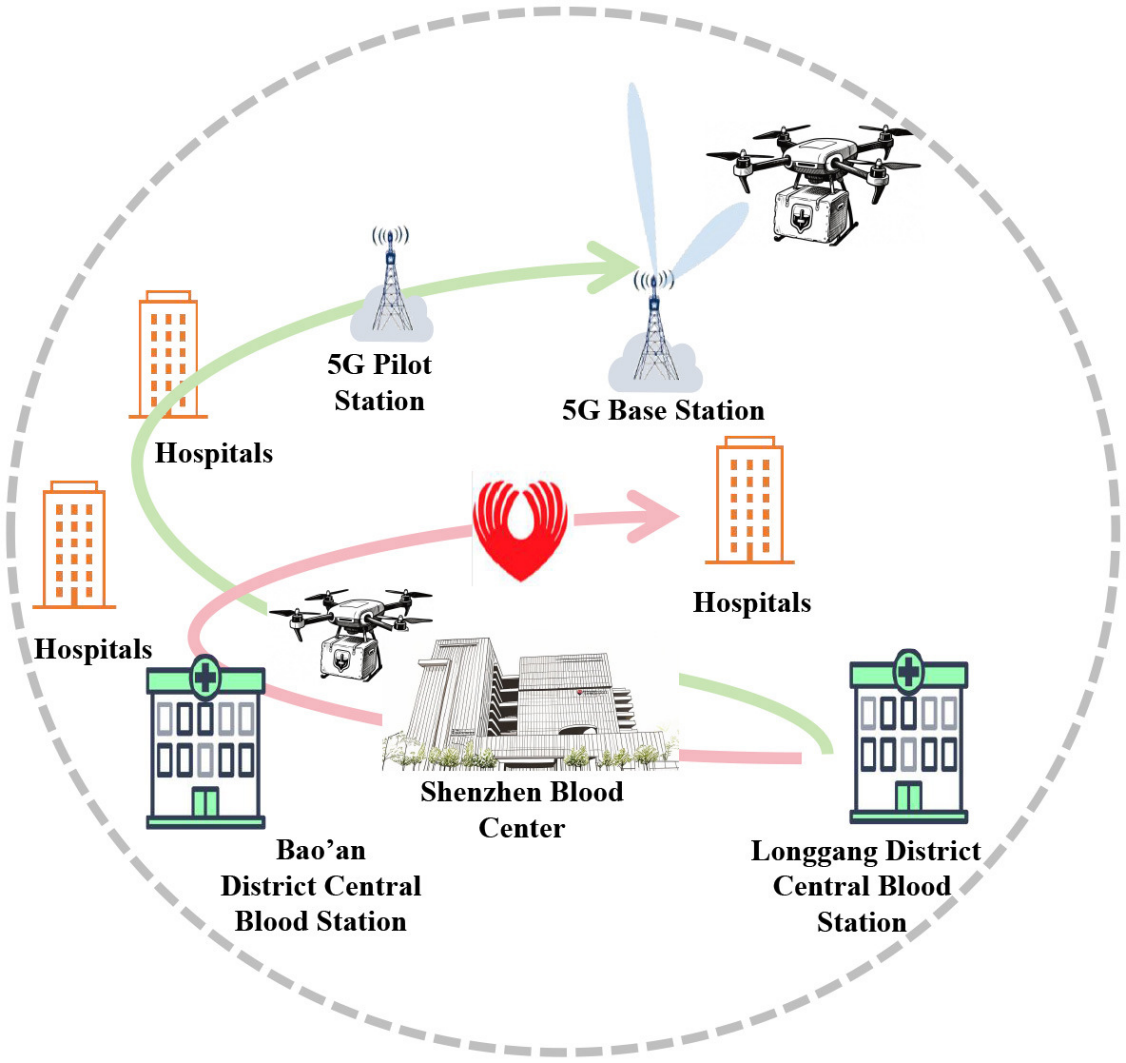
Shenzhen blood transport emergency support network. One blood center, in collaboration with two blood stations (Bao'an District Central Blood Station, Longgang District Central Blood Station), serves a network of multiple medical institutions.

## 4 Discussion

The management framework for drone blood transportation encompasses three major systems: flight dispatch, ground handling, and monitoring management. Among these, the flight dispatch system arranges and schedules drone sorties based on the urgency of demand, transportation distance, and the quantity of blood; the ground handling system is responsible for blood loading, and information reception; while the monitoring system undertakes the functions of ensuring transportation safety, monitoring and recording environmental parameters. Currently, there are two main management models for drone blood transportation: one is to entrust a single drone enterprise to handle blood transportation in a specific region, and the other is for blood collection and supply institutions to directly manage drone transportation operations. Both models have shortcomings, such as low participation from medical institutions as service recipients, poor integration of drone operations with the existing business of blood collection and supply institutions, and a lack of professional technical personnel and technological upgrade capabilities within these institutions to maintain normalized drone operations.

To address the above issues, this study proposes a 5G-enabled intelligent drone platform for blood transportation. This platform consists of hardware (takeoff and landing fields, Supplementary Figure 1) and software (management platform, Supplementary Figure 2), supports the integration of multiple drone enterprises, and enables unified dispatch. Its core advantages are reflected mainly in three aspects: first, open collaboration, allowing medical institutions to independently choose service providers and achieve customized transportation services; second, Fully automated integration, using AGVs (automated guided vehicle) to connect drones with smart cold storage facilities, enabling automated loading and emergency response; and third, intelligent management and control, leveraging 5G technology to monitor temperature control and flight status throughout the process, ensuring that the entire transportation workflow is controllable and traceable.

Despite the recent advancements exemplified by our proposed 5G platform, the global application of drone blood delivery, which is a typical application scenario of the low-altitude economy in the healthcare field, dates back to 2016 (18), still faces several universal challenges that need to be addressed.

Firstly, in terms of cost, although the time cost of drone blood transportation is lower than that of ground transportation (19), the overall expense of normalized transportation remains relatively high. Also in our study, compared with ground transportation, drone transport can save approximately 50% to 80% of time in congested traffic or remote areas, but the initial investment in equipment and systems is relatively high, and future research will need to address cost issues. In Shenzhen, the adoption of an open collaboration model, which allows medical institutions to independently choose service providers, has introduced competition among multiple enterprises. By introducing this competitive mechanism, it becomes possible in the future to reduce transportation costs through market forces, and the cost of drones may become more predictable and universal as the technology matures (20). In the long run, it will deliver significant social and economic value in emergency deployment and routine distribution.

Secondly, drone blood transportation in Shenzhen utilizes the city's public 5G network for remote drone control and data transmission, rather than building a dedicated network. It fully leverages existing communication infrastructure to ensure low-latency, high-reliability operation. However, in areas with insufficient 5G signal, real-time transmission of images and other data may be affected. Therefore, the coverage of drone blood transportation is limited in some smaller cities, as well as rural or mountainous regions. This coverage needs to be expanded to remote areas to improve emergency response efficiency, as international experts have proposed that drone-based blood delivery is one of the promising innovative strategies for blood transfusion in rural settings worldwide (7, 21).

Thirdly, technical bottlenecks need to be overcome, including further enhancing drone endurance, flight stability, and performance in adverse weather conditions. Under unfavorable weather, flight cancellation and alternative transport methods are initiated. Currently, drones have a maximum range of no more than 20 km, and long-endurance flights rely on battery replacement at relay points. As flight distance increases, the number of transfers rises, diminishing the time advantage. In this study, the maximum distance between the blood center and the hospital is 14.3 km, corresponding to a maximum flight time of approximately 21 min. The drone features a redundant battery design, ensuring safe flight and landing even in the event of a single power source failure, with a safe continuous flight time of 30 min. In the Shenzhen model, during initial route establishment, pre-assessments were conducted based on hospital preferences and blood usage specifics to optimize route selection. In the future, efforts should be directed toward technological innovation and development by advancing the research, application, and implementation of related technologies to increase payload capacity, enhance battery endurance, and extend flight range.

Finally, there are gaps in the legal framework, as drone blood transportation involves both aviation and medical fields. Establishing a sound regulatory system, formulating unified flight rules, and setting standards for drone blood transportation are urgent issues to address.

In our work, from January 1, 2024, to August 31, 2025, We recorded a total of 549 days, of which, normal operation days accounted for 57.92%. The flights of remaining days were grounded due to various reasons, such as airspace restrictions (27.69%), bad weather (10.75%), equipment maintenance (2.19%), college entrance exam impacts (1.09%), and equipment failures (0.36%; Table 6). It is evident that airspace management and weather conditions are the key factors affecting transportation regularity and should be prioritized for attention and improvement. According to our definition of adverse events, minor operational deviations such as brief delays, adjusting transport methods due to weather, holding in airspace and so on are not counted as adverse events, provided that the blood quality, clinical use and blood bag are not affected. As of August 31, 2025, the system has successfully completed a total of 11,784 flight sorties. All blood products have been delivered safely and on time, and the transport temperatures have remained stable, with temperature records fully compliant with the Blood Transportation Standard. WS 400−2023, and no abnormalities or damage occurred due to overheating or undercooling during blood transportation. The system operates with proven stability and reliability. In contrast, during ground transport, a small number of blood bags were rejected owing to breakage, leakage, or other causes. Statistical data showed that the wastage rate remained at 0.02% in both 2024 and 2025 (Supplementary Table 2).

**Table 6:** Overall summary of drone transportation status from January 1, 2024, to August 31, 2025.

Transportation status	Days	Percentage (%)
Normal flight	318	57.92%
Equipment maintenance	12	2.19%
Grounded due to weather	59	10.75%
Grounded due to airspace restrictions	152	27.69%
Grounded due to college entrance exam	6	1.09%
Grounded due to equipment failure	2	0.36%
Total	549	

However, there are still some limitations in this study. With respect to the temperature analysis, temperature records were generated for each drone blood transport, since all readings remained relatively stable overall and no impairment of blood quality due to temperature changes was observed, only a limited amount of temperature data was included in this study. The selected temperature records were obtained through stratified random sampling based on multiple dimensions, including time and route. We aim to intuitively and representatively demonstrate the stability of the temperature, not to highlight the sheer volume of the data. In the future, we will establish a continuous temperature quality assessment database. Moreover, Due to the high cost of the equipment and systems of drone transportation, and insufficient 5G signal will affect real-time transmission of images and other data, this study was conducted only in Shenzhen, with no corresponding studies in other cities especilly in specially rural/disaster areas.

Based on the advantages and challenges of drone blood transportation, future efforts will focus on the following tasks: expanding drone blood transportation routes to cover more medical institutions and reduce operational costs; coordinating with three blood collection and supply institutions in the city to establish aerial transportation and emergency channels, enabling rapid blood allocation via drones and building a city-wide, responsive aerial network for emergency blood transportation; and connecting street blood donation stations with blood centers to provide regular drone blood collection services, shortening transportation times; Conducting specimen transportation for clinical blood transfusion. The 5G-enabled intelligent drone platform for blood transportation establishes a new management model for blood collection and supply institutions. As an important application scenario for the development of the low-altitude economy in the healthcare industry, it holds significant importance for enhancing blood transportation capacity and ensuring emergency blood supply.

## Data Availability

The original contributions presented in the study are included in the article/Supplementary material, further inquiries can be directed to the corresponding authors.
