# Supplementary material for: 5G intelligent airport platform for UAV blood transport in Shenzhen China: a retrospective observational study
Source: Front Public Health. 2026 Sep 30;14:1947820. doi: 10.3389/fpubh.2026.1947820 (PMC13661936; doi:10.3389/fpubh.2026.1947820)
Supplement: Supplementary file 1 [file Supplementary_file_1.docx]

1. Construction of 5G-based Intelligent Airport Platform of Multi-rotor Drone

The 5G-based intelligent airport platform for drone blood transportation is equipped with advanced facilities and technologies, including drone takeoff and landing sites, intelligent dispatch rooms, real-time monitoring equipment, as well as an intelligent information management system based on 5G technology, which comprises an intelligent flight scheduling system and a monitoring management system. The core of the intelligent airport platform is built on the 5G network, featuring high bandwidth and low latency. It integrates 5G, automation, temperature control, and the Internet of Things (IoT), enabling it to resist interference in complex urban environments, maintain stable drone takeoff and landing, ensure fast real-time data transmission, and guarantee the safety and rapid transportation of blood.

(1) Hardware Facilities: Drone Takeoff and Landing Sites and Intelligent Dispatch Rooms

The multi-rotor drone takeoff and landing sites (SFig. 1) and intelligent dispatch rooms form the core hub of the intelligent airport platform for drone blood transportation, similar to traditional aviation airports. Currently, two drone companies have been stationed, and four drone takeoff and landing sites have been constructed, all of which are fixed sites capable of simultaneously launching multiple drones while ensuring the safety and stability via the 5G network. The drone model we used is the TR9S, which is specifically designed for medium-to-long-range transport of 15-20 kilometers, effectively connecting urban central blood stations with suburban hospitals. Additionally, the takeoff and landing sites require the following supporting facilities: power supply systems, protective fencing, monitoring systems, security code locks, nighttime lighting, explosion-proof battery charging cabinets, firefighting facilities, meteorological facilities, and cleaning and maintenance facilities and so on. The intelligent dispatch room is primarily used for aircraft placement, system scheduling management of the intelligent airport platform, and pilot stationed operations. It provides intelligent flight scheduling, establishing a dual-mechanism safeguard for routine blood transportation and emergency blood delivery. It also offers routine operational services for drone and route management. Furthermore, it interfaces with the blood collection and supply system to achieve closed-loop management of the entire process, from blood dispatch, loading, landing, to archiving.

**SFig. 1 Schematic diagram of the drone (left) and the take-off and landing site for 5G-based blood transportation (right)**

(2) Software Facilities: 5G-based Intelligent Information Management System

This management system is built on technologies such as 5G, big data, and artificial intelligence analysis. The software platform mainly consists of two modules: one for receiving blood demand, delivery planning, and transportation process management, and another one for end-to-end monitoring that integrates functions such as temperature monitoring, video surveillance, route tracking, flight status alerts, emergency dispatch, and hospital receipt confirmation. Through these modules, the platform achieves visibility, manageability, controllability, and traceability of drone blood transportation, constructing an integrated, intelligent, and open airport command and dispatch information management system to ensure blood quality and transportation safety. As a hub for drone blood transportation management, this intelligent airport platform can openly interface with blood collection and supply institutions across the city, forming a unified aerial blood transportation management system (SFig. 2).

**SFig. 2 Schematic Diagram of the Intelligent Airport Information Management Platform for Drones**

2. Drone Blood Transportation Process

Both the drone takeoff and landing site and the intelligent dispatch room are located at the blood center and operate on a 24-hour duty mechanism. When hospitals have blood demand, they can submit blood pre-orders via an online platform and opt for drone emergency delivery services. Upon receiving the pre-order notification, the blood center immediately initiates the response process, completing tasks such as blood pre-allocation, distribution, and packaging operations, after which the blood awaits drone loading and transportation.

**SFig. 3 Flowchart of Drone Blood Transportation Process**

3. Risk Control Measures in Actual Operations

(1) Airspace Conflict Management: Before each daily flight, the operators coordinate airspace usage with other airlines in advance to avoid time conflicts. If a potential delay is caused by airspace impact, the operations control center will evaluate the time efficiency and safety between waiting for airspace recovery and activating an alternative plan (e.g., ground transport), and select the optimal option.

(2) Route Management: Fixed routes are used for flights; except for special abnormal circumstances, no temporary route changes occur.

(3) Operational Approach Under Adverse Weather Conditions

Flight Cancellation Criteria: Level 6 wind (sustained wind speed > 10.7 m/s), heavy fog (visibility < 50 m), moderate rain (precipitation > 0.42 mm/h), and severe weather warnings such as thunderstorms and hail. If the weather forecast issued before the flight indicates conditions exceeding the safety threshold, the operators immediately switch to ground transport to avoid delays.

Emergency Alternative Mechanism: Once a flight is canceled, the dispatch center notifies the backup land transportation to carry out the transport mission.

Decision-Making Responsibilities: The duty operations manager is responsible for integrating real-time meteorological data and warning information to make the final decision on whether to proceed with the flight.

4. Hospital-Side Landing Areas

Each receiving hospital is equipped with a standardized fixed landing platform, typically located on a building’s rooftop or in an open area within the hospital grounds. The platform is equipped with prominent markings, nighttime lighting, and safety guardrails. The landing zone is connected to the hospital’s blood transfusion department via a dedicated passage, enabling the handover of blood products within 10 minutes of the drone’s arrival. This forms an end-to-end closed-loop logistics system.

**SFig. 4 Standardized fixed landing platform of the hospital.**

**Table1 The aerial distances, ground distances, approximate ground transport times, and drone transport times between the distribution center and the 15 hospitals**

|  | aerial distance (Km) | road distance(Km) | approximate road travel time (min) | UAV travel time (min) | Time saving (%) |
| --- | --- | --- | --- | --- | --- |
| hospital 1 | 12.8 | 15.0 | 32.9 | 19.7 | 40.1 |
| hospital 2 | 10.0 | 11.6 | 28.2 | 16.7 | 40.8 |
| hospital 3 | 12.9 | 14.0 | 27.4 | 22.2 | 19.0 |
| hospital 4 | 13.5 | 16.0 | 32.1 | 18.6 | 42.1 |
| hospital 5 | 11.2 | 12.0 | 22.8 | 14.0 | 38.6 |
| hospital 6 | 9.6 | 11.0 | 22.6 | 13.2 | 41.6 |
| hospital 7 | 27.5 | 36.7 | 42.8 | 36.1 | 15.7 |
| hospital 8 | 30.2 | 38.3 | 64.4 | 39.6 | 38.5 |
| hospital 9 | 28.6 | 36.3 | 45.9 | 37.7 | 17.9 |
| hospital 10 | 20.9 | 23.3 | 42.3 | 28.5 | 32.6 |
| hospital 11 | 2.9 | 4.2 | 12.9 | 5.1 | 60.5 |
| hospital 12 | 3.7 | 5.4 | 17.8 | 6.0 | 66.3 |
| hospital 13 | 11.1 | 10.5 | 27.5 | 14.9 | 45.8 |
| hospital 14 | 26.6 | 32.3 | 57.1 | 35.4 | 38.0 |
| hospital 15 | 7.9 | 10.2 | 23.0 | 12.0 | 47.8 |

| Year | Number of blood bags transported | Number of damaged bags | Wastage rate (%) |
| --- | --- | --- | --- |
| 2024 | 291,650 | 62 | 0.02 |
| 2025 | 260,744 | 52 | 0.02 |

**Table 2 Blood wastage rate of ground transport**
